# Researchers’ perceptions of malaria eradication: findings from a mixed-methods analysis of a large online survey

**DOI:** 10.1186/s12936-020-03430-2

**Published:** 2020-10-08

**Authors:** Joe Brew, Menno Pradhan, Jacqueline Broerse, Quique Bassat

**Affiliations:** 1 Barcelona Institute for Global Health, Hospital Clinic, c/Rosselló, 132, 5è 2a, 08036 Barcelona, Spain; 2 Vrije Universiteit Amsterdam, De Boelelaan 1105, 1081 HV Amsterdam, Netherlands; 3 University of Amsterdam, REC E, Roetersstraat 11, Amsterdam, Netherlands; 4 Centro de Investigação em Saúde de Manhiça (CISM), Maputo, Mozambique; 5 Institució Catalana de Recerca i Estudis Avançats, Pg. Lluís Companys 23, 08010 Barcelona, Spain; 6 Pediatric Infectious Diseases Unit, Pediatrics Department, Hospital Sant Joan de Déu (University of Barcelona), Barcelona, Spain; 7 Consorcio de Investigación Biomédica en Red de Epidemiología y Salud Pública, Madrid, Spain

**Keywords:** Malaria, Eradication, Elimination, Mixed methods, Survey, Crowdsourcing, Probability, Opportunity cost

## Abstract

**Background:**

The value of malaria eradication, the permanent reduction to zero of the worldwide incidence of malaria infection caused by human malaria parasites, would be enormous. However, the expected value of an investment in an intended, but uncertain, outcome hinges on the probability of, and time until, its fulfilment. Though the long-term benefits of global malaria eradication promise to be large, the upfront costs and uncertainty regarding feasibility and timeframe make it difficult for policymakers and researchers to forecast the return on investment.

**Methods:**

A large online survey of 844 peer-reviewed malaria researchers of different scientific backgrounds administered in order to estimate the probability and time frame of eradication. Adjustments were made for potential selection bias, and thematic analysis of free text comments was carried out.

**Results:**

The average perceived likelihood of global eradication among malaria researchers approximates the number of years into the future: approximately 10% of researchers believe that eradication will occur in the next 10 years, 30% believe it will occur in the next 30 years, and half believe eradication will require 50 years or more. Researchers who gave free form comments highlighted systemic challenges and the need for innovation as chief among obstacles to achieving global malaria eradication.

**Conclusions:**

The findings highlight the difficulty and complexity of malaria eradication, and can be used in prospective cost–benefit analyses to inform stakeholders regarding the likely return on eradication-specific investments.

## Background

Malaria is a parasitic disease transmitted among humans by mosquitoes. *Plasmodium falciparum* accounts for many of the 200 million annual cases as well as most of the half million annual deaths worldwide [1, 2]. Malaria “elimination”, the “interruption of all local transmission of the infection in a country or region” [3] is actively being pursued by dozens of countries around the world, leading to a renewed push for “eradication” (“the permanent reduction to zero of the worldwide incidence of malaria infection caused by all species of human malaria parasites”) [4]. The rationale for pursuing eradication is straightforward: the annual burden of malaria is so high that eradication is simply “necessary” [5]. With this renewed push has come renewed debate on whether a time frame is a realistic or desirable component of eradication [4].

This is not the first time that malaria eradication has been in the international spotlight. The scientific and public health communities have had eradication on their policy agenda since the World Health Organization (WHO) established the Global Malaria Eradication Programme in the 1950s [6]. In 1957, U.S. President Dwight Eisenhower told Congress that malaria could be expected to be eradicated in five years. Despite an “extraordinary sense of international purpose”, the top-down and one-size-fits-all campaign which followed did not achieve its goal [7]. Following the failure of the WHO’s first attempt, the focus shifted away from global eradication towards local control strategies, though the goal of global eradication was never formally abandoned. The change in strategy from global eradication to local control had the effect of less interest and funding for aggressive anti-malarial interventions, leading to an increase in malaria’s burden. In recent years, much of the discourse regarding malaria has shifted back to global eradication [8], with funders, researchers, and public health practitioners rallying to the cause [3]. The Bill and Melinda Gates Foundation began actively promoting eradication beginning in 2007, and in recent years has described eradication as feasible “within a generation” [9]. The WHO also set ambitious goals, stating the objective of eliminating malaria in 35 new endemic countries from 2015 through 2030, and reducing all deaths from malaria by 90% [10]. Progress towards elimination and eradication efforts slowed, however, towards the end of the decade. The WHO Strategic Advisory Group on Malaria Eradication acknowledged “stalling progress” and that meeting the 2015 targets was “unlikely” [11]. Meanwhile, the Lancet Commission argued that global eradication by 2050 was both a “necessary” and “attainable goal” [5].

From both policy [12] and scientific [13] points of view, eradication has never before received so much attention. Prior to the Lancet Commission, most recent research on expert opinion regarding the feasibility of malaria eradication focused on the *how* rather than the *if* and *when* [3]. International programmes, such as the WHO Global Malaria Programme (GMP), have acknowledged the need “to take an official position on how and under what timeline malaria eradication could be achieved” [14]. The Lancet Commission did just this, setting 2050 as the mark. However, the WHO Strategic Advisory Group on Malaria Eradication argued that “eradication by a specific date is not a promise we can make” [11]. In other words, there seems to be universal consensus among experts regarding the desirability of global eradication, but discord on the timeline and feasibility.

Clarity on timeline and likelihood of eradication could inform forecasting of disease transmission, and plays a crucial role in the economic analysis of the expected value of malaria eradication initiatives, ultimately informing health policy decisions. But achieving clarity is difficult, given the many complex and interacting variables, which affect malaria transmission, research funding, and technological development. The lack of a clear scientific consensus regarding the timeline to and probability of eradication can be considered an important knowledge gap with real consequences: not knowing an “investment’s” maturity date or risk profile is a deterrent to any “investor” (public or private) in the public good of malaria eradication. In other words, funding for eradication-oriented interventions could potentially be greater if funders perceived less uncertainty. Similarly, policy-makers in resource-scarce contexts must weigh the hypothetical benefits of a costly intervention against the risk of the intervention’s intended outcome not occurring; having more clarity on eradication’s timeline and likelihood could inform these decision-making processes.

The general objective of eradication serves to inspire, rally funder support, motivate researchers, and focus the efforts of public health practitioners. To the extent that malaria eradication (by definition) has never occurred, the parameters needed for an ex post cost–benefit analysis are unknown. Nonetheless, prospectively estimating eradication’s return on investment is crucial to deciding when and how to pursue the goal, especially in light of the high direct and opportunity costs of eradication-specific interventions. One approach to economic evaluation is the use of infectious disease transmission models. These have been applied to diseases which are closer to eradication than malaria, since the uncertainty around model input parameters is less, requiring fewer cascading assumptions in order to present possible comparative scenarios. For example, Kastner et al*.* were able to describe 4 relatively realistic pathways to lympathic filariasis eradication, as well as the pre-requisite role and magnitude of certain interventions [15]. A similar modelling framework was then used to estimate the cost of eradication [16]. A recent modelling analysis on onchocerciasis eradication, based on a disease transmission model, showed that the costs of elimination (relative to staying in “control mode”) in Africa would be far lower even in the short term, thanks to the improvements it would lead to in both treatment times and prevented surveillance costs. Given the relative proximity of eradication, and the narrow geographic scope, the authors were also able to estimate the timeline to eradication [17].

This level of detail and specificity in the economic evaluation landscape of malaria eradication, unfortunately, is not possible, given its high prevalence and epidemiological complexity. In fact, there are no transmission models estimating the likelihood and time frame of eradication, or its derivative cost–benefit ratio. Globally, where transmission modelling has been used for the purposes of forecasting the future epidemiology of malaria, the methods have generally been aimed at optimizing elimination methods, determining whether a strategy is scalable [18], guiding funding and drug development [19, 20], or comparing a range of hypothetical morbidity scenarios [21], rather than assessing the likelihood of or time until the occurrence of eradication.

To the extent that estimating eradication’s likelihood and timeframe is essential to forecasting the cost–benefit ratio of eradication interventions, alternative methods are needed to forecast such parameters. Just as the stock market aggregates perceptions to provide an assessment of something as complex as a company’s value, aggregating perceptions may be a useful tool for tackling the complexity of malaria eradication. Many studies have shown value in expert elicitation as a means to reduce uncertainty and inform decision-making [22], and various techniques—such as the well-known Delphi Technique—exist to generate consensus from multiple points of view [23]. As Francis Galton demonstrated in his famous study in which he showed that the crowds’ aggregated estimates of cow’s weight formed a quasi-normal distribution centred around the true weight [24, 25], averaging the perceptions of many can be more accurate than taking the opinion of any single expert, since the biases of diverse viewpoints can be complementary and symbiotic. Measuring consensus and discord among disease-specific researchers from a variety of disciplines can serve as a barometer of (informed) opinion, both guiding resources and identifying areas of concern [26].

The optimal assignment of resources for malaria eradication campaigns hinges on the expected value of those campaigns, the latter being a direct function of the discounting applied to future benefits and the probability of “success” (i.e., eradication). Holding constant factors such as the cost of eradication and the benefits of achieving it, the return on investment of malaria eradication initiatives is a function of eradication’s timeframe (assuming a > 0 discount rate) and probability (assuming a < 100% likelihood of success). Given this, estimating these parameters is crucial to evaluating if and when attempts at eradication should be undertaken.

The aggregation of malaria researchers’ perceptions regarding the time frame and likelihood of eradication can be understood to form a probability distribution, which can be used to estimate the expected value of eradication-specific investments. The objective of this study is to gauge (expert) opinion about, estimate the likelihood of, and quantify the potential time frame to malaria eradication through a systematic online survey of malaria research professionals from a wide array of academic disciplines. Doing somay guide the optimal distribution of health resources by informing estimations of the expected value of malaria eradication efforts.

## Methods

The study population included all first authors (with available email addresses) returned in a PubMed search for the term “malaria” from January 1, 2010 through December 20, 2016. PubMed was used because it was the most comprehensive publication database for malaria-related research, and also exposed enough metadata about articles and authors so as to allow for relevant analysis. Personalized emails addressing the author by name and mentioning the relevant paper were sent to each of the 7680 authors during the period from December 20, 2016 through January 2, 2017. Researchers were invited to participate by clicking a link to the survey form. The survey was simple, consisting of only name, email, and four content-related fields along with a “general comments” section.

Content-related survey fields consisted of:

1. Area of expertise.
2. Perceived probability (%) of malaria eradication in 10, 20, 30, 40, and 50 years.
3. Free choice perceived number of years until malaria eradication.

The survey was intentionally as short as possible, so as to appeal to time-pressed participants. However, supplementary data on researchers is useful for the assessment of selection bias and determinants of perception, we estimated participant gender, total number of citations, and total number of peer-reviewed articles published. In order to estimate whether a user was male or female, data were used from the North Atlantic Population Project, and U.S. government [27]. Total citations and total publications were binned into three categories: 0–5 (junior-level researchers, PhD students); 6–99 (most professional academics); and > 99 (the most prolific researchers). The searching and retrieval of information pertaining to articles and citations from the PubMed database was carried out using tools from the RISmed package [28]. Citations and publications outside of the PubMed database were not captured. Information retrieved about authors was used to de-bias parameters in an ordinary least squares regression, analysing the association between number of publications and perceived years to eradication.

Survey results were first analysed descriptively. Following Galton’s example [25], point estimates for event probabilities were estimated as the average of all responses, and the totality of the responses to each numeric question were used to estimate uncertainty around those likelihoods and time frames.

Quantitative analysis was carried out in R language (R Core Team, 2015). Qualitative analysis of free text comments was carried out using thematic analysis, with inductive open coding carried out iteratively [29]. In the final, open-ended question, participants were invited to provide “any general comments on the timeline and likelihood of global eradication”. Thematic analysis [30] was employed to code responses following the 6-phase approach laid out by Braun and Clarke [31]. The approach underwent several iterations in which codes were modified, discarded and created. One coder carried out all thematic coding and classification; quality control was assured through multiple, iterative reviews with the authorship team. Using the RQDA software to assist in data management and theme coding [32], four subject themes were identified. Comments were additionally coded as either descriptive (comments pertaining to the “problem” of malaria eradication) or prescriptive (pertaining to potential “solutions” for eradicating malaria). Finally, free-text comments were scored for overall sentiment polarity [33].

## Results

### Participant characteristics

A total of 884 researchers participated in the survey from the 7918 invitations sent (participation rate of 11.2%). Areas of expertise were non-exclusive and self-described, with participants having the option to choose from up to 3 of 10 checkboxes, or to write in one or more “other” areas of expertise. 604 (68.3%) participants declared at least one area of expertise.

Participants had a total of 219 unique (self-described) areas of expertise. The five most popular were Epidemiology (357), Information Technology (344), Parasitology (319), Biology (277), Clinical medicine (207) (see Table 1).

**Table 1:** Sample size and average perceived years until eradication by area of expertise

Area of expertise	Average years	Number of participants
GIS	50	5
Infectious disease	50	7
Malaria	50	8
Medical entomology	49.09	12
Political science	48.75	14
Vector control	48.75	9
Drug discovery	48	5
Microbiology	48	5
Pharmacology	47.78	9
Anthropology	47	20
Economics	46.84	34
Public health	45.96	29
Entomology	45.84	58
IT	45.79	344
Parasitology	45.74	319
Biology	45.70	277
Virology	45.65	23
Clinical medicine	45.56	208
Epidemiology	45.31	357
Immunology	44.3	104
Bioinformatics	44	5
Statistics	43.73	86
Ecology	42.57	8
History	42.5	6
Pharmacy	42	5
Vector biology	41.6	5
Geography	40	5
Chemistry	37.46	27
Biochemistry	37	5
Medicinal chemistry	34.57	7

Values of more than 50 years or “never” were coded as 50 for the purpose of estimating averages

Respondents were qualitatively different from non-respondents. Importantly, the average number of total author-specific citations was 40.9 among respondents, but 92.9 among non-respondents. When examining the number of average citations per article, the difference between respondents remained: 4.8 among respondents, and 9.0 among non-respondents, highlighting the greater impact of non-respondents relative to respondents. Males responded at a greater rate (12.2) than females (9.1).

### Perceptions of likelihood of eradication

**Fig. 1:**
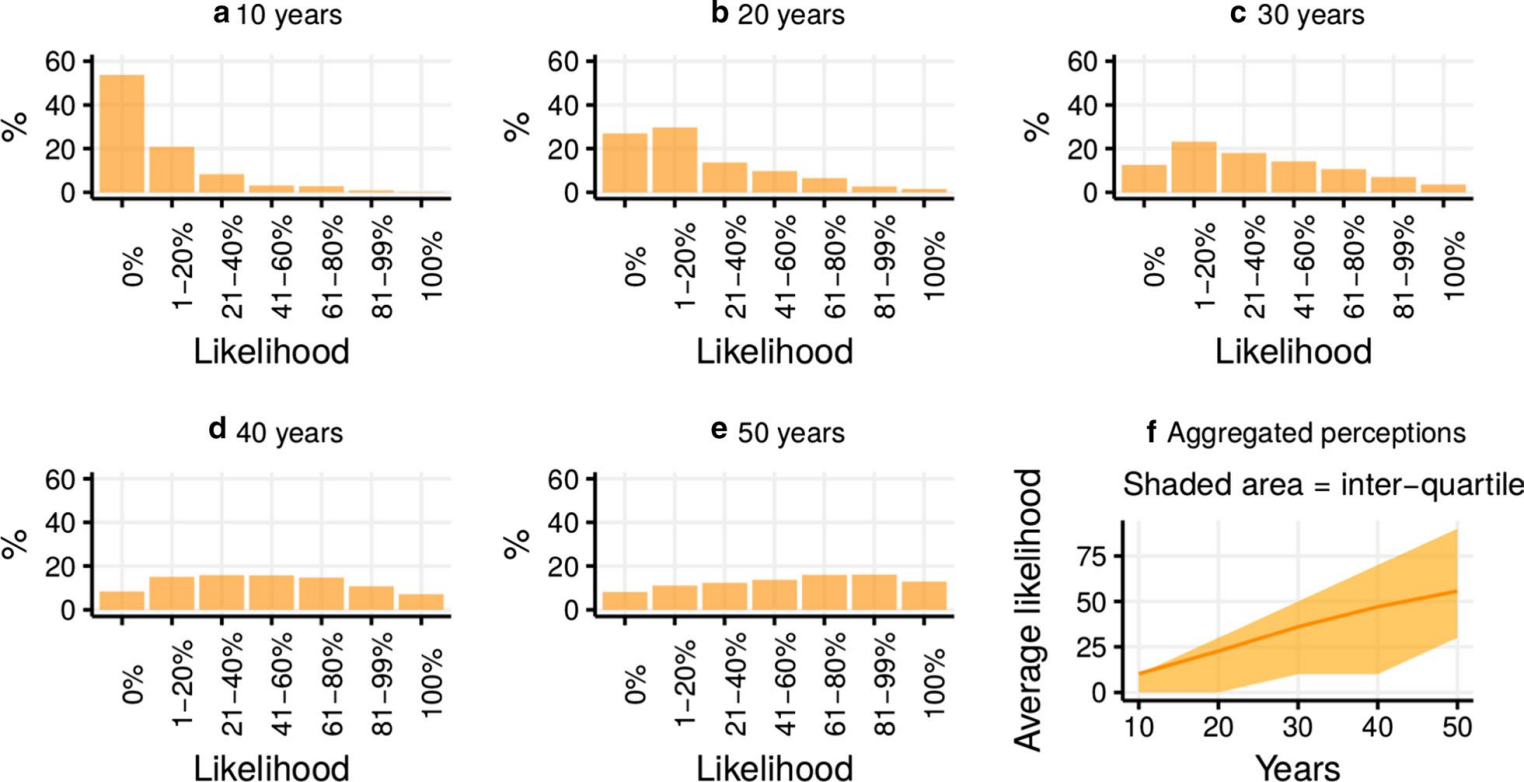
Perceptions of likelihood of eradication

Most participants saw eradication as extremely unlikely in the next 10–30 years, but increasingly likely thereafter. Figure 1 shows the distribution of year-specific likelihood perceptions (panels A–E), as well as an illustration of how both likelihood and uncertainty grow over time (panel F). At the 40-year mark, the distribution of perceived likelihood of eradication appears “normal”, and by 50 years it is slightly shifted to the right (i.e., consensus is towards eradication more probable than not).

In regards to responses to perceived years until eradication, 59 (0.7%) were either blank or unintelligible, whereas 825 participants provided an estimated number of years. Three quarters of the respondents, 616 (74.7%) estimated that it would be 50 or more years until eradication. Differences were not significant between different areas of expertise (Table 2).

**Table 2:** Response rate by participant characteristics

Variable	Characteristic	Responded	Invited	Response rate (%)
Sex	Female	209	2287	9.1
	Male	358	2939	12.2
	Unknown	317	2692	11.8
Citations	0–5	621	3299	18.8
	6–99	179	3270	5.5
	> 99	84	1349	6.2

**Fig. 2:**
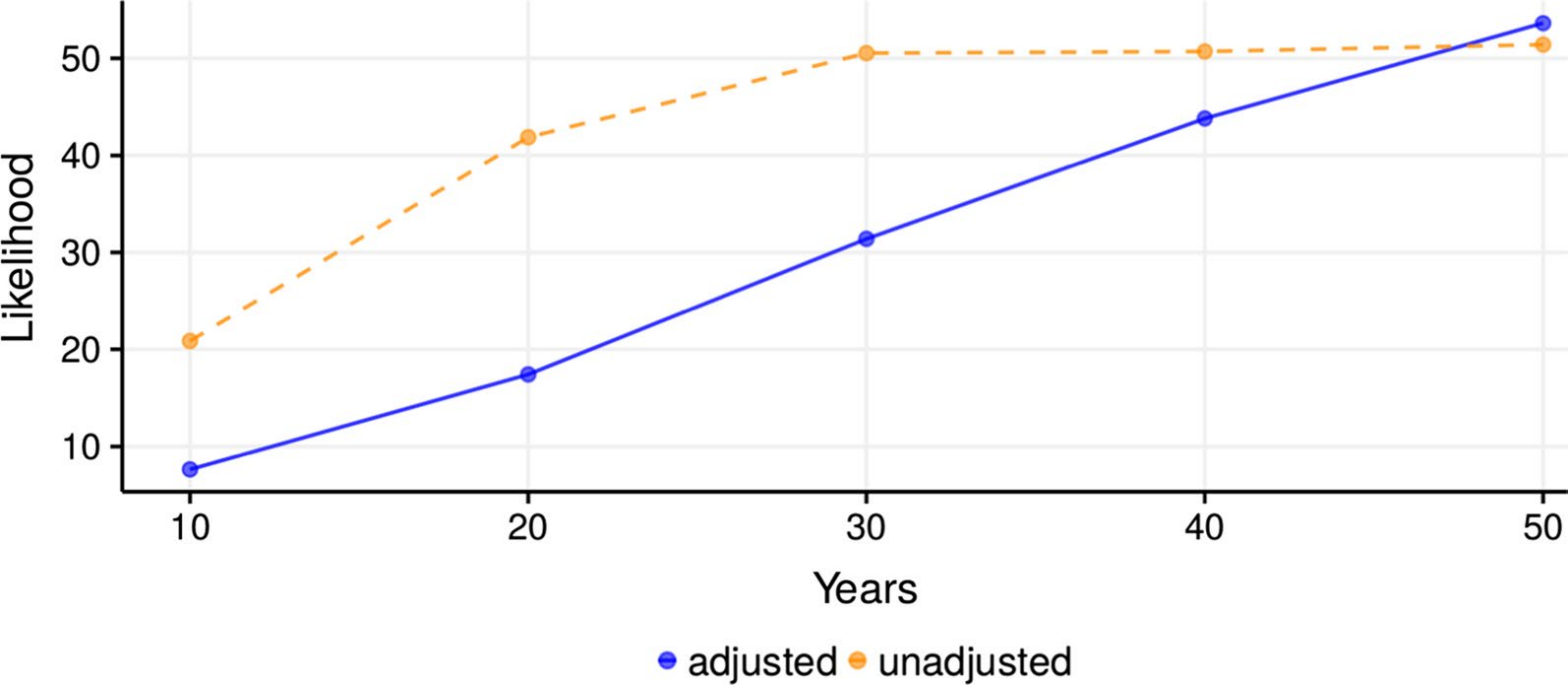
Aggregated perceived likelihood of eradication over time, adjusted for sample selection and unadjusted

In order to de-bias sample selection, a simple binomial logistic regression model was estimated on the likelihood of response as a function of gender and (binned) number of citations. Having estimated the odds of survey participation, the inverse of the selection model’s predictions were employed as weights in a simple linear model to adjust estimates. A separate weighted model estimated the likelihood of eradication at 10, 20, 30, 40, and 50 years. Figure 2 shows both the aggregated perceived likelihood of eradication over time before and after adjusting for sample selection based on binned number of publications and gender. Adjusted and unadjusted estimates coincide in the long-term, but diverge sharply in the near-term; adjustment suggests that had the pool of respondents been more representative, the average perceived likelihood of near-term eradication would have been much lower. Roughly, the adjusted perceived likelihood of eradication tracks the number of years into the future.

### Perceptions of eradication’s challenges and complexity

Of the 884 who responded to the survey, 540 (61.1%) provided a comment. Relative to non-commenters, commenters were more optimistic on average, but also more polarized in opinion. The three subject themes identified through the procedure of iterative, open coding were:

1. *Solutions*: Comments pertaining to the innovations required to achieve eradication, priorities, and the desirability of certain approaches.
2. *Systemic challenges*: comments pertaining to political, social, environmental or logistical issues related to eradication.
3. *Complexity*: comments which focus on the multi-dimensional components of eradication.

Comments were also classified as descriptive or prescriptive. A majority (59.3%) were descriptive. Descriptive commenters were more pessimistic (in regards to perceived years until eradication) than prescriptive commenters, though this difference did not reach the level of statistical significance (p = 0.21, Pearson’s Chi-square). Descriptive comments also received sentiment polarity scores which were more negative than prescriptive comments, although again this difference did not reach the level of statistical significance (p = 0.18).

In regards to *solutions*, comments largely pertained to the necessity of further technological advances and innovations. One clinical epidemiologist wrote that “currently available technology can’t achieve [eradication], even if delivered optimally”; a parasitologist argued that eradication could not be achieved without a “game-changing innovation”, whereas multiple others referred to the need for “transformative” technologies:

*We can't achieve eradication with our current tools. We'd need new medicines, a better vaccine, and maybe other vector control tools.*

Many noted the need to “overcome the challenge of drug resistance”. More than 10% of commenters noted the need for an effective vaccine. Genetic engineering was mentioned by several commenters as a promising means to achieve eradication quickly. Most comments coded as “solutions” were prescriptive in nature, often suggesting the nature of the needed innovation, with a heavy slant towards pharmaceutical options and vaccination.

*Systemic challenges* to malaria eradication were noted by the majority of commenters. Comments in this category can be divided into four sub-themes: (i) lack of coordination, (ii) lack of good surveillance and health services delivery, (iii) lack of political will and (iv) poverty. In direct contrast to the previous comments, many echoed the comments of an epidemiologist who stated that “we already have the tools to achieve eradication” and that the only piece lacking was “robust health systems”. Many commenters noted problems of coordination, as illustrated in the below quote from a biologist.

*It will be very difficult to eradicate malaria… not because we don't have the technologies, which we already have… the problem is politics. Malaria doesn't stop in (sic) borders of a country and it would take a joint effort of a lot of political leaders to get programs in place to fight malaria. Unfortunately I don't see this happening anytime soon.*

Others echoed the sentiment, with many comments focusing on the need for strong surveillance and treatment delivery systems. Many commenters focused on other reasons for stagnating progress; for example, a public health specialist pointed out the importance of “weak or failing health systems…due to political unwillingness or conflict”. Many noted that malaria is a “disease of poverty”, with “social injustice” as the root cause. Some made the sequential argument that “eradication of poverty” must precede disease eradication. Along the same lines, one epidemiologist wrote:

*Eradication requires a full systems-wide approach, not a disease-specific approach. The eradication of smallpox was a triumph of management, not medicine or technology.*

Another epidemiologist noted that the survey “left off the list the most important factor—economic development”. Many echoed the sentiment, stating that without economic development, eradication will be impossible, and that poverty is the “cause” of malaria. Comments coded as “systemic” tended to be descriptive and more pessimistic than others.

*Complexity* was a relatively rare category (< 20% of all comments), those whose comments were coded as the “complexity” category were more pessimistic than average in regards to the timeline and likelihood of eradication. Many commenters highlighted the inherent challenges in the epidemiology of malaria, such as the changing dynamics of malaria transmission, the resilience of the parasite and vector, climate change, and the inability to aim interventions accurately with an “ever-moving target”. The potential for adaptation was highlighted in reference both to the mosquito as well as the parasite itself. Several participants pointed out that the vast animal reservoirs for *Plasmodium knowlesi* made it "impossible to eradicate". Many comments addressed the fact that the conversation on eradication is largely taking place within the public health community, whereas the causes of malaria endemicity are largely orthogonal to public health interventions. Several commenters pointed out the multitude of prerequisite conditions for eradication to even be considered feasible; for example, an economist and statistician noted:

*To my mind, this question is highly dependent on background context, e.g. global political and economic dynamics, as well as international conflict. Complete global eradication is an extremely singular goal that requires a vast array of necessary conditions - if any of these fail, eradication will not be achieved.*

Many commenters thought that the terms “eradication” and “malaria” were so complex and nebulous that public health practitioners should avoid them all together, so as to not repeat the mistakes of the WHO GMP in the 1950s. For example, some implied that there were—at least operationally—multiple "malarias", and talking about “malaria” as one disease misses the mark, since the different species of parasite and contexts in which they live make elimination in each area very distinct from other areas. One clinician wrote that eradication is a “postwar” idea that developed from the “abandonment of a broad sociopolitical understanding of the causes of disease, and the emphasis on technological solutions.” Many stated that global malaria eradication was simply not possible, and two argued that it may not be desirable or ethical. One epidemiologist stated that the concept was “absurd” and that “I’m not even sure why people talk about it”. A clinician questioned the utility of discussing “eradication” as a concept:

*Eradication is a different objective than elimination. Elimination means that the disease is not endemic but could reappear even in a country like Norway if infrastructure breaks down. Elimination may be possible in poor endemic countries, following socioeconomic development. Eradication means that the parasite disappears from the planet, which is not realistic…*

Comments questioning the utility of eradication as a concept or goal tended to be skeptical of its feasibility. Largely, they were prescriptive, advocating for a re-framing of the conversation so that the focus was not on an “arbitrary” goal like eradication, but rather on scaling up control and making region-specific progress.

## Discussion

This study has elicited researchers’ view, through an online survey, on likelihood and approximate time to malaria eradication. Roughly three-quarters of respondents believe that malaria will not be eradicated in the next half century. When adjusted for selection bias, the perceived likelihood of eradication in 50 years remains similarly low, but estimates for shorter-term eradication are even lower.

Eradication of a disease is a “high stakes game”, exposed to multiple competing—and at times contradictory—incentives from multiple stakeholders [34]. Understanding these incentives, and the factors which can alter them, is vital to anticipate how eradication can succeed, and the specific aspects where failure may be more likely. In fact, disease eradication is a typical example of public good, where free-riding can determine failure. Cooperation, collaboration, generation of incentives or potentially even impositions could represent solutions to the free-riding problem. Either in the absence or with not so stringent budget constraints limiting the investment, a high and certain return on investment could, by itself, constitute a key disincentive to free ride. However, the return on investment of eradication-specific interventions is affected by the fact that most researchers agree that eradication will take a long time to achieve. This, in turn, reduces the expected value of future benefits, disincentivizing eradication-specific investment. Given this, it is important to quantify the positive externalities of “failed” eradication, as well as the potential backfire effects. That is, the reduction in burden of disease can still make interventions worthwhile, but an abandonment of efforts if ambitious goals are not met may lead to resurgence in malaria as was the case in the 1970s following the GMEP’s failure at eradication. This study did not endeavour to make this quantification.

However, areas of high malaria endemicity are often also those with high competing health costs. When estimating the return on investment of malaria elimination initiatives, not only must one take into account the potential benefits (even in the case of failure), but also the opportunity costs (even in the case of success). After all, eradication and elimination are not binary success/failure propositions—an initiative should be judged both on its epidemiological circumstances [35] as well as the counterfactual improvements in health which could have been achieved via other paths. Even if one considers malaria eradication to be as utopic as "world peace", it might still have utility (in terms of generating political momentum and mobilizing resources), as well as risks.

## Limitations

This paper has several limitations. Conceptually, academic researchers are specialists—their narrow, field-specific view of eradication’s feasibility is of arguable reliability, given that they may be unfamiliar with the operational, cultural and “real-world” challenges of malaria eradication. Though crowds have been found to be more “wise” than individuals in many cases, the application of an approach similar to the “wisdom of crowds” is not suitable to all classes of problems [36]. Crowds can be susceptible to social biases [37] (although this survey’s anonymity largely protects against this issue), and other biases may come in to play, especially given that our study was of a crowd of “specialists” (from only one publication database), rather than the population as a whole.

Though sample size was large, response rates were low, calling for the need to address potential selection bias. In the case of gender, even though males responded at a significantly greater rate than females (p < 0.001, Pearson’s Chi-squared), selection bias was not of concern since there were not significant differences by gender in regards to pessimism/optimism (ie, time frame or likelihood of eradication). In the case of researcher impact (as measured by the total number of citations), selection bias plays an important role: being highly-cited was associated both with eradication “pessimism” as well as likelihood of non-response. In other words, the pool of respondents was less highly-cited than the pool of invitees, and among respondents, those who were highly-cited tended to be more pessimistic. Though results were de-biased, selection bias on other (unobserved) variables may still exist.

Three additional potential biases are worth mentioning specifically. (1) “Conjunction fallacy” suggests that the general goal of eradication may seem less likely than the aggregation of the goals of country-specific elimination. (2) A (reverse) variant of the “hot hand fallacy”, in which researchers may mistakenly base their assessment of current chances of eradication on previous failures. (3) Parkinson’s law of triviality suggests that researchers may disproportionately see the challenges of their own research (e.g. anti-malarial drug resistance) as larger or more relevant to the global eradication campaign than they really are.

As with the survey of experts of Keenan et al*.,* regarding the feasibility of eradication of neglected tropical diseases, this study detected relatively high levels of eradication skepticism and did not delve into whether researchers had clinical or operational experience, nor did it assess opinions of program workers, nor did it explore complexities pertaining to different types of or forms of existence of malaria. Though this study’s sample size was over twice Keenan’s, this was largely due to having contacted more authors, as response rate was only one fourth as large [26].

This study included the first authors of indexed journals. Though certainly a group with important knowledge related to malaria, this misses malaria control programme employees, health agency workers, and other stakeholders. Their experiences and viewpoints may be different from those of academics, and arguably more relevant. The de-biasing method accounts for different response rates of “senior” vs. “junior” researchers, but does not take into account the fact that first authors are generally more junior than senior authors (i.e., the pool from which samples came may have been biased itself). To the extent that the results suggest that those with less experience (as represented through publications) tended to be more “optimistic” regarding eradication, it is reasonable to assume that the restriction of first authors may have led to an overly optimistic sample, making the results of the survey even more striking. Given these issues, the extent to which the respondents are representative of malaria experts cannot be known with certainty.

There are other important limitations which may also affect this study’s generalizability. The restriction of the source of data to only the PubMed platform meant not detecting researchers who publish in journals indexed elsewhere. Data was collected in late 2016 and early 2017, and the extent to which the opinions of participants have changed is unknown. Though the qualitative coding of free-text comments was reviewed by all authors, only one individual carried out the coding. This, along with the low response rate among invited participants, suggests that this study may have been subject to both selection and analysis bias; results should be understood within this context.

## Conclusion

The findings of this survey show researchers expressing hesitance about the likelihood of eradication and suggesting a long-time frame until it is achieved. The causes for skepticism are diverse, but common themes were the need for innovation, systemic challenges, and the complexity of the disease and its transmission.

The implication of these results are twofold: (1) that those working or investing in eradication-specific campaigns, as well as those modeling these campaigns’ hypothetical cost–benefit, should factor in researchers’ perceived long time frame when calculating those campaigns’ expected value; (2) that champions of near-term eradication may need to make a more compelling case to malaria researchers of eradication’s feasibility, in order to better focus and inspire the latter.

The “true” feasibility and timeframe of eradication is unknown, as only time will tell whether the collective “wisdom” of researchers was worth adhering to or not. The actual cost–benefit of eradication interventions is not only a function of eradication’s success, but also of a number of other factors which are only knowable retrospectively. This study’s primary contribution is the provision of a snapshot of perceptions of malaria researchers, whose opinions may be of value not only to other researchers, but also to the malaria and public health communities at large.

## Data Availability

The datasets analysed during the current study are available from the corresponding author upon reasonable request.

## Abbreviations

GMEP: Global Malaria Eradication Programme
GMP: Global Malaria Programme
WHO: World Health Organization
